# Successful Sequential Treatment From Remdesivir to Molnupiravir for Prolonged COVID-19 in a Patient With Follicular Lymphoma and Renal Pelvic Carcinoma: A Case Report

**DOI:** 10.7759/cureus.75722

**Published:** 2024-12-15

**Authors:** Tomoki Satoh, Kentaro Akata, Kei Yamasaki, Akimasa Tsuruta, Kazuhiro Yatera

**Affiliations:** 1 Department of Respiratory Medicine, University of Occupational and Environmental Health, Japan, Kitakyushu, JPN; 2 Division of Infection Control and Prevention, University of Occupational and Environmental Health, Japan, Kitakyushu, JPN

**Keywords:** molnupiravir, prolonged covid-19, sars-cov-2, sars-cov-2 infection, subsequent antiviral therapy

## Abstract

A 69-year-old Japanese male with follicular lymphoma and renal pelvic carcinoma presented with fever and cough, testing positive for SARS-CoV-2 via PCR. Chest CT revealed ground-glass opacities. Initially, his symptoms improved with a 10-day course of remdesivir (RDV), but they recurred. On day 42, a second 10-day course of RDV combined with dexamethasone was initiated; however, symptoms persisted, and his SARS-CoV-2 PCR test remained positive on day 72. Subsequently, a successful treatment regimen of 10 days of RDV followed by five days of molnupiravir (MOL) was administered. This study represents the first reported case of persistent SARS-CoV-2 infection successfully treated with sequential therapy transitioning from RDV to MOL, without extending the treatment duration.

## Introduction

The World Health Organization declared the end of the COVID-19 pandemic as a global health emergency on May 5, 2023; however, COVID-19 continues to circulate in the population. Consequently, the reduction of infection control measures may increase the risk of a future rise in infections.

Another risk factor is prolonged viral shedding in patients with persistent SARS-CoV-2 infections, particularly among immunocompromised individuals [1-3]. Among patients with cancer, those with hematological malignancies frequently experience prolonged SARS-CoV-2 persistence [4]. This phenomenon is especially pronounced in individuals undergoing B-cell depletion, such as those treated with anti-CD20 antibodies or those receiving hematopoietic stem cell transplantation or cellular therapy within the past year, as well as those with chronic lymphopenia [5]. B cells are critical for eliminating viruses through humoral immunity, which may explain the persistence of SARS-CoV-2 in these patients. Such prolonged infections can delay the timely initiation of essential treatments for underlying conditions, including anticancer therapies and surgeries. Moreover, immunosuppressed patients with SARS-CoV-2 may act as reservoirs for mutated strains that are challenging to treat [6].

Currently, the optimal therapeutic strategy for individuals experiencing extended viral shedding beyond the duration of initial treatment remains unclear. We present a case of persistent SARS-CoV-2 infection in a patient with follicular lymphoma and renal pelvic carcinoma who was successfully treated with sequential antiviral therapy transitioning from remdesivir (RDV) to molnupiravir (MOL). This report is significant as it details the use of subsequent antiviral therapy without extending the treatment duration.

## Case presentation

We present the case of a 69-year-old Japanese male with prolonged persistence of SARS-CoV-2 who had follicular lymphoma and renal pelvis carcinoma. The patient had no history of vaccination against SARS-CoV-2. His lymphoma was diagnosed in 2020 and treated with obinutuzumab and bendamustine, followed by 12 cycles of rituximab maintenance therapy, resulting in complete remission (the date of the final dose was April 2023). In June of the same year, he was diagnosed with renal pelvic carcinoma, and treatment with gemcitabine and carboplatin was initiated.

**Figure 1:**
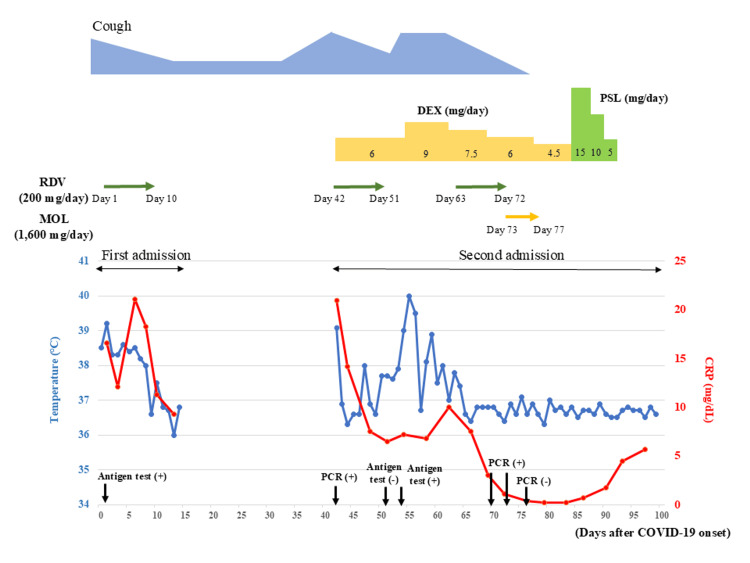
Clinical course of the patient The course of cough symptoms is indicated in the blue area. The duration of RDV administration is marked with green arrows, while the duration of MOL administration is indicated by yellow arrows. Body temperature and serum CRP levels are represented in blue and red, respectively. CRP, C-reactive protein; DEX, dexamethasone; MOL, molnupiravir; PSL, prednisolone; RDV, remdesivir

**Figure 2:**
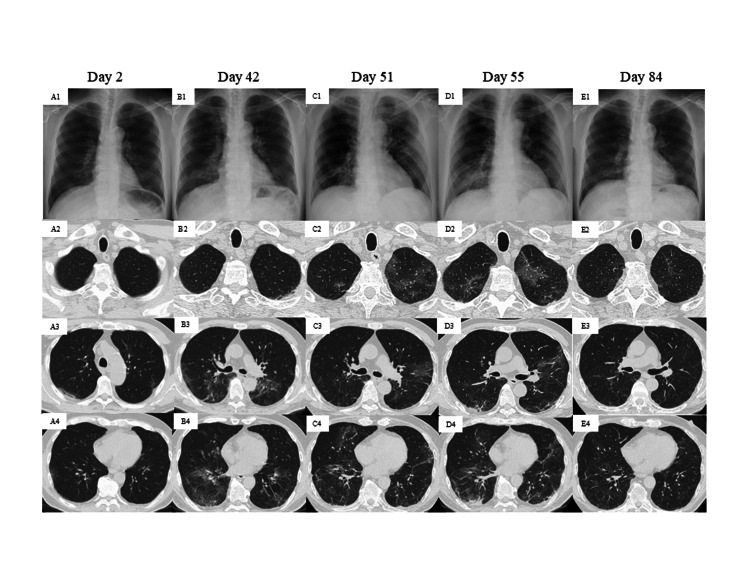
Radiographic progression of the disease on chest X-ray and CT Chest X-ray and CT findings revealed subtle subpleural GGOs in both upper lung fields on day 2 after the onset of coronavirus disease 2019 (A3). By day 42, bilateral GGO appeared in both lower lung fields (B1-B4). On day 51, GGO was observed in the left upper (C2) and lingula lung fields (C3), accompanied by a reduction of GGO in both lower lung fields (C4). On day 55, subpleural consolidation and relapse of GGO in the right lower lung fields were noted (D1-D4). By day 97, these pulmonary opacities had nearly disappeared (E1-E4). GGO, ground-glass opacity

He received a second course of gemcitabine and carboplatin in September 2023, and a week later, he developed a fever and cough. The following day, he tested positive for SARS-CoV-2 antigen (ImunoAce® SARS-CoV-2 Ⅱ) on a nasopharyngeal swab for the first time (day 1; the first day of onset of COVID-19) (Figure 1). His chest CT demonstrated subtle ground-glass opacities (GGOs) in both upper lungs (Figure 2 A3). A 10-day course of RDV (200 mg/day) was initiated, and granulocyte colony-stimulating factor was administered from days 2 to 4 in response to grade 4 neutropenia after 10-12 days of chemotherapy. The cough slightly improved on day 7, followed by a subsidence of fever on day 9, and he was discharged on day 14. After discharge, he remained afebrile but had a persistent cough.

In late October, he manifested symptoms of fever and exacerbated cough. His chest radiograph demonstrated hazy opacities in both lower lung fields (Figure 2 B1), and his CT revealed diffuse GGO in both lower lung fields on day 42 (Figure 2 B2, B3, and B4). His nasopharyngeal swab was positive for SARS-CoV-2 on the BioFire FilmArray Respiratory Panel 2.1 (RP2.1) multiplex PCR test (bioMérieux, Inc., Marcy-l’Étoile, France), and he was readmitted to our hospital. A 10-day course of RDV and dexamethasone (DEX) (6 mg/day) was initiated, followed by the resolution of fever the next day. He tested negative for the SARS-CoV-2 antigen on day 51, and his chest GGO also tended to improve (Figure 2 C1-C4). However, three days after his second admission, he redeveloped fever and cough exacerbations. Another antigen detection test for SARS-CoV-2 was positive on day 54.

The chest radiograph demonstrated consolidations in his right lower lung field (Figure 2 D1), and chest CT showed subpleural consolidation with worsening GGO in the lower right lung field (Figure 2 D2, D3, and D4). The DEX dose was increased from 6 mg to 9 mg daily. Because fever and cough persisted along with a sustained elevation of C-reactive protein levels (Figure 1, Table 1), a 10-day course of RDV was resumed on day 63. Despite the alleviation of fever and cough, the SARS-CoV-2 PCR remained positive on day 72. Considering the risk of recurrence, MOL (1,600 mg/day) was subsequently administered for five days following RDV treatment. The patient tested negative on PCR on day 76, remained afebrile, and chest images on day 84 showed improvement in the GGO and consolidation (Figure 2 E1-4). Corticosteroid tapering persisted and was concluded on day 93; he was clinically stable and discharged on day 99. Subsequently, the patient had no recurrence for three months.

**Table 1:** Laboratory findings on admission Alb, albumin; ALT, alanine aminotransferase; APTT, activated partial thromboplastin time; AST, aspartate aminotransferase; BUN, blood urea nitrogen; Cre, creatinine; CRP, C-reactive protein; FDP, fibrin/fibrinogen degradation products; Hb, hemoglobin; Ht, hematocrit; LDH, lactate dehydrogenase; Plt, plate; PT, prothrombin time; PT-INR, prothrombin time-international normalized ratio; RBC, red blood cell; T-bil, total bilirubin; TP, total protein; WBC, white blood cell; γ-GTP, gamma-glutamyl transferase

Parameter	Level	Reference
WBC (× 10^3^ μL)	5.6	3.3-8.6
Neutrophils (%)	78	38.0-74.0
Lymphocytes (%)	14	16.5-49.5
Eosinophils (%)	1	0.0-8.5
Monocytes (%)	7	2.0-10.0
Basophils (%)	0	0.0-2.5
RBC (× 10^6^ μL)	4.74	4.35-5.55
Hb (/µL)	14.8	13.7-16.8
Ht (%)	42	40.7-50.1
Plt (× 10^4^ μL)	15.1	15.8-34.8
PT-INR	1.04	0.94-1.15
APTT (seconds)	24.9	24.0-39.0
FDP (μg/mL)	10.6	0.0-5.0
D-dimer (μg/mL)	2.5	0.0-1.0
Fibrinogen (ng/mL)	828	200-400
TP (g/dL)	5.5	6.6-8.1
Alb (g/dL)	2.2	4.1-5.1
T-bil (mg/dL)	0.4	0.4-1.5
AST (U/L)	26	13-30
ALT (U/L)	27	10-42
LDH (U/L)	401	124-222
γ-GTP (U/L)	43	13-64
BUN (mg/dL)	16	8-20
Cre (mg/dL)	1.43	0.65-1.07
Na (mEq/L)	132	138-145
K (mEq/L)	4.2	3.6-4.8
Cl (mEq/L)	95	101-108
Ca (mg/dL)	7.8	8.8-10.1
CRP (mg/dL)	21.03	0.00-0.14

## Discussion

We report a case of a patient with persistent SARS-CoV-2 infection successfully treated with sequential therapy transitioning from RDV to MOL for the first time, with no recurrence observed over three months. This subsequent therapy may represent an optimal option for managing persistent SARS-CoV-2 infections in immunocompromised patients.

Most cases of persistent SARS-CoV-2 infection can be effectively treated with standard therapies; however, in immunocompromised patients, particularly those with hematological and non-hematological malignancies, antiviral treatment may be challenging if the viral load is not sufficiently reduced by initial antiviral agents [7,8]. In particular, the use of immunosuppressive medications, such as corticosteroids, baricitinib, and/or tocilizumab, for patients with COVID-19 requiring oxygen therapy or mechanical ventilation may lead to chronic refractory viral infections due to immunosuppression [9].

To date, three types of antiviral treatments have been reported to reduce viral load in patients with persistent SARS-CoV-2 infection. These include nirmatrelvir/ritonavir (NMV/r) therapy [10,11], combination therapy comprising RDV and NMV/r [10,12,13], NMV/r combined with MOL [14], and RDV combined with ensitrelvir [15]. Additionally, sequential therapy transitioning from RDV to NMV/r has been documented [10], although most of these treatments involved extended durations, except for one report featuring combination therapy [15] (Table 2).

**Table 2:** Summary of reported cases of persistent COVID-19 successfully treated with combination therapy, switch therapy, and/or therapy with extended duration ALL, acute lymphoblastic leukemia; CLL, chronic lymphocytic leukemia; DLBCL, diffuse large B-cell lymphoma; FL, follicular lymphoma; MCL, mantle cell lymphoma; MOL, molnupiravir; NMV/r, nirmatrelvir/ritonavir; RDV, remdesivir; TSH, thyroid-stimulating hormone

Case	Author	Age/sex	Country	Type of hematological malignancy	History of vaccination	Duration from onset to start of successful treatment	Antiviral drug				Use of steroid	Adverse effects of antiviral drug
							Type of antiviral drug (administration period)	Combination therapy	Switch therapy	Therapy with extended duration		
1	Trottier et al. (2023) [12]	64/M	USA	CLL	+	About four months	RDV+NMV/r (20d)	+	-	+	+	None
2	Ford et al. (2023) [13]	40/M	USA	B-cell ALL	-	About four months	RDV (10d)+NMV/r (20d)	+	-	+	+	Abdominal bloating
3	Breeden et al. (2023) [10]	79/F	USA	FL	+	About six months	NMV/r (21d)	-	-	+	-	None
4	Breeden et al. (2023) [10]	72/M	USA	DLBCL	+	About one month	RDV (10d)+NMV/r (20d)	+	-	+	-	None
5	Breeden et al. (2023) [10]	72/M	USA	MCL	+	About three months	RDV (10d)→NMV/r (15d)	-	+	+	-	None
6	Breeden et al. (2023) [10]	60/F	USA	B-cell ALL	+	About two months	RDV (7d)→NMV/r (21d)	+	+	+	-	None
7	Marangoni et al. (2023) [14]	73/M	Italy	FL	Unknown	About two months	NMV/r+MOL (10d)	+	-	+	+	None
8	Liu et al. (2023) [11]	34/M	USA	B-cell ALL	+	About six months	NMV/r (15d)	-	-	+	-	None
9	Liu et al. (2023) [11]	55/M	USA	B-cell ALL	+	About five months	NMV/r (18d)	-	-	+	-	Transient transaminitis and elevated TSH levels
10	Jung et al. (2023) [15]	59/F	Japan	FL	+	About one month	RDV (3d)+ensitrelvir (5d)	+	-	-	-	None
11	Present case	69/M	Japan	FL	+	About two months	RDV (10d)→MOL (5d)	-	+	-	+	None

Although all these treatments were successful and reported no side effects, aside from two cases involving mild abdominal bloating [12] and mild transient transaminitis with slightly elevated thyroid-stimulating hormone levels [11], clinical questions remain regarding their general applicability. Further information is needed due to a lack of sufficient evidence concerning their efficacy and safety. Additionally, the safety of NMV/r administration beyond 10 days remains unproven for extended durations [16].

Regarding the duration of RDV treatment, previous research indicated no significant difference in clinical improvement between five-day and 10-day courses of RDV in patients with COVID-19 pneumonia who did not require mechanical ventilation [17]. However, the subjects in that study had mild comorbidities, such as diabetes, hyperlipidemia, hypertension, and asthma, without severe immunodeficiency. Thus, a five-day RDV course may not achieve a sufficient reduction in viral load compared to a 10-day course in patients with severe immunodeficiency.

Based on this information, a 10-day course of RDV was administered to our patient from days 63 to 73. However, the SARS-CoV-2 PCR test remained positive, with a cycle threshold (Ct) of 33 on day 72. Following RDV treatment, the patient exhibited a reduction in fever and cough, and the high Ct indicated a low viral load, suggesting partial clinical improvement. Nonetheless, given the patient’s severely immunocompromised state due to follicular lymphoma, renal pelvic carcinoma, and long-term corticosteroid use, there remained a significant risk of viral load escalation if antiviral treatment was discontinued. Therefore, a five-day course of MOL was initiated from days 73 to 77, resulting in a negative SARS-CoV-2 PCR test on day 76 and successful treatment without recurrence. In this case, the sequential use of MOL following RDV may have resulted in a cumulative antiviral effect, ultimately leading to viral clearance. For severely immunocompromised patients with SARS-CoV-2 who continue to test positive after an RDV regimen, it may be advisable to consider MOL as a follow-up therapy.

A limitation of this study is that the genotypes of the virus during the first and second hospitalizations were not determined; therefore, it is possible that our patient was infected with different SARS-CoV-2 subtypes. However, we hypothesize that the COVID-19 episodes occurring during separate hospitalizations could be attributed to a persistent SARS-CoV-2 infection, as the patient’s cough continued after discharge. Another limitation is that this is a single case report; thus, the true efficacy of the current therapies remains inconclusive. Further research with a larger number of cases is necessary to confirm these findings.

## Conclusions

We report the first case of a patient with persistent SARS-CoV-2 infection successfully treated with sequential therapy transitioning from RDV to MOL without extending the treatment duration. While further research and clinical trials are necessary, this sequential therapy may represent an optimal approach for managing persistent SARS-CoV-2 infections.
